# Return to Work Within 104 Weeks in Working-Age Patients Following Total Hip Arthroplasty

**DOI:** 10.7759/cureus.104934

**Published:** 2026-03-09

**Authors:** Nicky T Wouters, Karolina A. P. Wijnands, Bert Boonen, Danny Steijvers, Jasper Most, Chantal L. I. Gielen, Martijn G. M. Schotanus

**Affiliations:** 1 Department of Social Medical Affairs, Dutch National Institute for Employee Benefits Schemes (UWV), Heerlen, NLD; 2 Department of Orthopedic Surgery, Zuyderland Medical Center, Sittard-Geleen, NLD; 3 Department of Research, Academic Knowledge Center Work and Health South-East Netherlands (AKAG-ZON), Heerlen, NLD; 4 Department of Social Medicine, Care and Public Health Research Institute (CAPHRI), Faculty of Health Medicine and Life Science, Maastricht University, Maastricht, NLD; 5 Department of Orthopedic Surgery, Care and Public Health Research Institute (CAPHRI), Faculty of Health Medicine and Life Science, Maastricht University, Maastricht, NLD; 6 Department of Epidemiology and Public Health, Care and Public Health Research Institute (CAPHRI), Faculty of Health Medicine and Life Science, Maastricht University, Maastricht, NLD

**Keywords:** occupational physician, occupational rehabilitation, postoperative outcomes, return to work, rtw, sex differences, social factors, social security, tha, total hip arthroplasty

## Abstract

Background

Total hip arthroplasty (THA) is increasingly performed in working-age adults, making return-to-work (RTW) an important outcome. In the Netherlands, RTW is particularly relevant due to the legal obligation for employers to cover up to two-years of sick leave. While short-term RTW outcomes after THA have been described, evidence on long-term RTW and its determinants remains limited. This study evaluated RTW up to two-years after THA and explored clinical, occupational, and sociodemographic factors associated with RTW.

Methods

This single-center cohort study included patients aged 18-65 years who underwent primary THA between January 2016 and February 2018 at Zuyderland Medical Center (Sittard-Geleen, the Netherlands). Eligible patients were invited to complete a questionnaire on employment status, RTW timing, occupational characteristics, expectations, and perceived facilitators and barriers, alongside patient-reported outcome measures (PROMs: Oxford Hip Score and EuroQol Five Dimensions (EQ-5D)). Descriptive analyses were performed, and multivariable logistic regression was used to identify predictors of RTW. Time to RTW was analyzed using reversed Kaplan-Meier curves.

Results

Of 381 eligible patients, 182 responded (53.8%), and 145 completed all questionnaires (mean age 58.7 ± 5.6 years; 51% female). Preoperatively, 66% were employed and 6% self-employed. Full RTW was achieved by 77% of employed and 78% of self-employed patients, with a mean time to RTW of 7.4 ± 4.4 weeks. Most patients RTW within 12-weeks, with additional RTW occurring up to 52-weeks postoperatively. Full RTW was most common in administrative, managerial, and care-related occupations, whereas physically demanding jobs more often required modified duties or reduced hours. Social support at work and job satisfaction were the most frequently reported facilitators of RTW, while physical complaints and high physical workload were the main barriers. In multivariable analysis (n = 106), female sex was the only independent predictor of non-RTW (odds ratio (OR) 3.74, 95% confidence interval (CI) 1.29-10.84, p = 0.015). Other clinical and occupational factors were not significantly associated with RTW.

Conclusion

The majority of working-age patients returned to work within 12-weeks after THA and remained employed for two years. Female sex was independently associated with a lower likelihood of RTW. Enhanced occupational guidance, workplace adjustments, and targeted support may further optimize sustainable work reintegration after THA.

## Introduction

Globally, osteoarthritis (OA) is one of the leading causes of global disability of the musculoskeletal system [1]. The incidence of OA is expected to rise even further, partly as a result of aging and an increasing incidence of obesity [2]. For people who do not benefit from non-invasive therapy, a total hip arthroplasty (THA) could be indicated to improve their quality of life [3]. Every year, the number of THAs being done continues to increase. In 2014 in the Netherlands, 28,194 primary THAs were performed, rapidly rising to 36,684 in 2023, which is predicted to continue growing [4].

In recent years, there has been an increase in the number of young patients undergoing THAs. The proportion of THA patients under the age of 65 years is expected to increase to half of all THAs performed by 2030 [5]. Combined with the increasing retirement age in the Netherlands, which expanded more than four years in the past decade, more working patients will likely need THA [6].

THA is recognized for its beneficial influence on health-related quality of life. Early return to daily activities, including return to work (RTW), is significant for younger patients [7]. To date, only brief outcomes have been documented, with a maximum one-year follow-up after THA addressing RTW [8,9]. A recent study found that the RTW rate after one year was only 70% [10]. Additional findings suggest that psychosocial working conditions, including support from occupational physicians (OPs) and supervisors, play a beneficial role in promoting early RTW [9,10]. However, little is known about long-term RTW in patients undergoing THA, which is of significant importance in the Netherlands. In the Dutch system, the employer is legally obligated to cover the costs of the first two years of sick leave under the gatekeeper act. Patients who have been on sick leave for 104 weeks are eligible to apply for a disability pension from the government [11]. The right to a disability pension is assessed based on the theoretical loss of income (earning capacity) due to the limitations as a result of the underlying disability. If the theoretical loss of earning capacity is more than 35%, a disability pension is granted after two years of sickness absence.

Understanding the long-term RTW following two years of inability is crucial. Therefore, this study aimed to examine the RTW of patients within two-years following THA. Furthermore, patients' socioeconomic status (SES), quality of life, experiences, and various factors influencing RTW were studied.

## Materials and methods

Patients and methods

**Figure 1:**
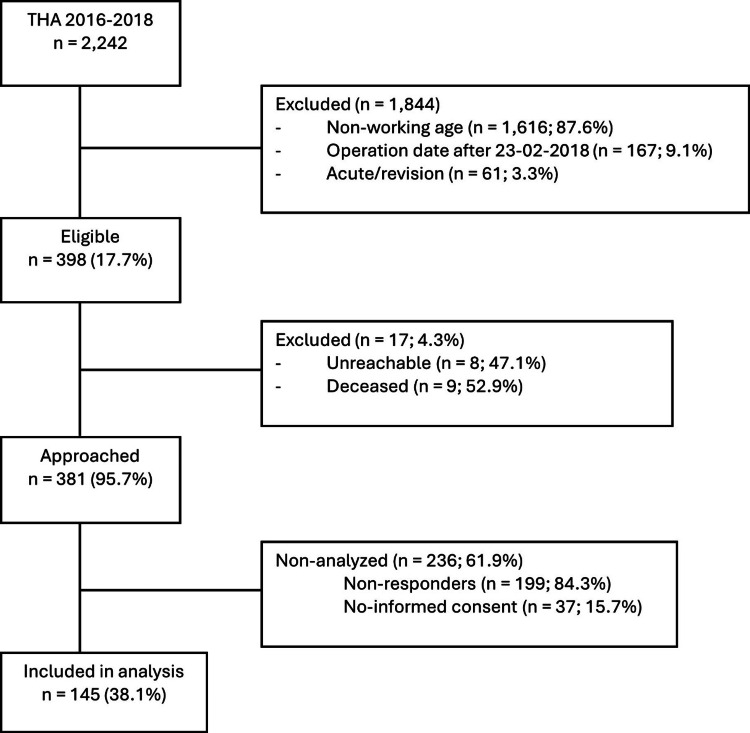
Flow chart of patient inclusion. THA: total hip arthroplasty

The present single-center cohort study included a consecutive series of patients operated on THA at the Zuyderland Medical Center (Sittard-Geleen and Heerlen, the Netherlands) between January 2016 and February 2018. In total, 2,242 THA were performed between January 2016 and February 2018 by experienced orthopedic surgeons or senior residents under supervision. All patients aged between 18 and 65 were eligible candidates for this trial. Patients with incomplete data registration in the patients’ records or with revision arthroplasty surgery were excluded from participation. In October 2020, eligible patients (n = 398, 17.8%) were approached to participate in this trial. Patient distribution is illustrated in Figure 1.

Data sources

Patient characteristics (e.g., age at initial THA, sex, body mass index (BMI; kg/m^2^), and American Society of Anesthesiologists (ASA) classification [12] were collected from the patient’s electronic medical data files. The four-digit postal code was used to estimate the SES according to data from the Dutch national Central Bureau for Statistics and the Social Cultural Planning office (CBS) [13]. SES was reported descriptively to provide context for the cohort but was not included as a predictor in the multivariable analysis due to limited variation within the sample.

Evaluation

A previously used RTW questionnaire [14] was administered, in which patients reported their pre- and postoperative employment status and whether they had RTW. The type of work was categorized into six groups: retired, light physical work, heavy physical work, administrative work, household work, and no job/looking for a job. Employed patients provided additional information on RTW expectations (Appendix A), working hours, and factors that facilitated or impeded RTW. Time to RTW was reported in weeks, and occupations were classified according to the Research Centre for Education and the Labour Market-Statistics Netherlands (ROA-CBS) 2014 classification of work [15].

SES was determined based on the postal code available from the CBS [13]. The SES is expressed as a standardized continuous score derived from data on the mean income percentage per household, education level, and labor market participation. The average SES score for the Netherlands is approximately 0; higher scores indicate residents are more prosperous and/or better educated and/or have been working longer.

To assess clinical results, patients received two different patient-reported outcome measures (PROMs): the Oxford Hip Score (OHS; 0 to 48; 48 best possible) [16] and the EuroQol-5D, consisting of two subdivisions: the index score (EQ-5D-index) that comprises five dimensions: mobility, self-care, usual activities, pain/discomfort, and anxiety/depression (-0.329 - 1.000; 1.000 is the best score). Patient’s self-rated overall health status was assessed with a Visual Analogue Scale (EQ-5D-VAS; 0 to 100; 100 being the best outcome) [17].

Approval from the medical ethics review committee was obtained before the study began (METCZ20200127). All included patients gave written consent.

Data analyses

All data were managed in Microsoft Excel (Microsoft Corp., Redmond, WA, USA) and analyzed using IBM SPSS Statistics for Windows, Version 29 (Released 2022; IBM Corp., Armonk, New York, United States). Continuous variables were assessed for normality using the Kolmogorov-Smirnov test. Normally distributed variables are presented as mean ± standard deviation (±SD), and non-normally distributed variables as median with interquartile range (IQR). Categorical variables are reported as frequencies and percentages (%). Baseline characteristics were summarized descriptively. Differences between groups were tested using one-way ANOVA (F) with least significant difference (LSD) post-hoc analysis for normally distributed continuous variables, and the Mann-Whitney U test (H) for non-parametric data. Categorical variables were compared using Pearson’s chi-square test (χ^2^).

Qualitative data from free-response questions were systematically analyzed to identify common themes related to factors influencing RTW, information resources regarding RTW, and causes of (partial) unemployment.

Multivariable logistic regression was performed to examine predictors of RTW, including treatment modality and other clinically relevant variables, adjusting for differences between patient groups. A reversed Kaplan-Meier analysis was used to evaluate time to RTW in weeks. A two-sided p-value < 0.05 was considered statistically significant.

## Results

Patient cohort

Of 381 patients approached, 182 (53.8%) responded, and 145 completed the questionnaire and PROMs. The average time between surgery and completing the questionnaire was 4.2 ± 0.8 years. The cohort had a mean age of 58.7 ± 5.6 years, 51% were female, and the mean BMI was 28.4 ± 4.6 kg/m^2^. Age differed significantly between employment groups (p = 0.003), while sex, BMI, ASA classification, SES, and most PROMs did not. Postoperative EQ-5D index differed between groups (p = 0.042). Patient characteristics and PROMs are summarized in Table 1 and Appendix B.

**Table 1:** Patient demographics and PROMs of employed, self-employed and non-employed patients. BMI: Body mass index; ASA: American Society of Anesthesiologists classification; SES: socioeconomic status; OHS: Oxford Hip Score; EQ-5D: EuroQol Five Dimensions; VAS: Visual Analogue Scale The data were represented as mean ± standard deviation. One-way analysis of variance (ANOVA) (F) was performed for comparing groups for continuous outcomes, and the chi-square test (Chi^2^) was used for comparing groups for categorical outcomes. P-value was considered significant if p < 0.05.

Variable	Employed			Self-employed			Non-employed			P-value	F/Chi^2^
	N	Mean	SD	N	Mean	SD	N	Mean	SD		
Age (years)	96	57.5	±5.8	9	61.3	±4.6	40	60.6	±4.6	0.003	5.946
Sex (% female)	48	50	-	2	28.5	-	24	60	-	0.115	4.317
BMI (kg/m^2^)	96	28.6	±4.7	9	27.8	±5.1	40	27.9	±4.4	0.700	0.358
ASA classification											
ASA I (%)	27	28.1	-	5	55.6	-	10	25	-	0.247	5.422
ASA II (%)	66	65.6	-	3	33.3	-	29	72.5	-		
ASA III (%)	6	6.3	-	1	11.1	-	1	2.5	-		
SES	96	-0.06	±0.19	9	0.01	±0.16	38	-0.07	±0.21	0.531	0.635
OHS (post)	96	38.0	±9.9	9	42.8	±7.6	40	37.1	±10.1	0.338	1.094
EQ-5D index (pre)	96	0.740	±0.060	9	0.762	±0.041	40	0.767	±0.045	0.145	1.983
EQ-5D index (post)	96	0.829	±0.270	9	1.000	±1.000	40	0.731	±0.360	0.042	3.249
EQ-5D VAS (pre)	96	58.6	±20.4	9	67.5	±5.0	40	57.4	±19.1	0.637	0.453
EQ-5D VAS (post)	96	77.3	±15.9	9	84.9	±10.1	40	76.5	±15.0	0.325	1.132

Employment status and RTW

**Figure 2:**
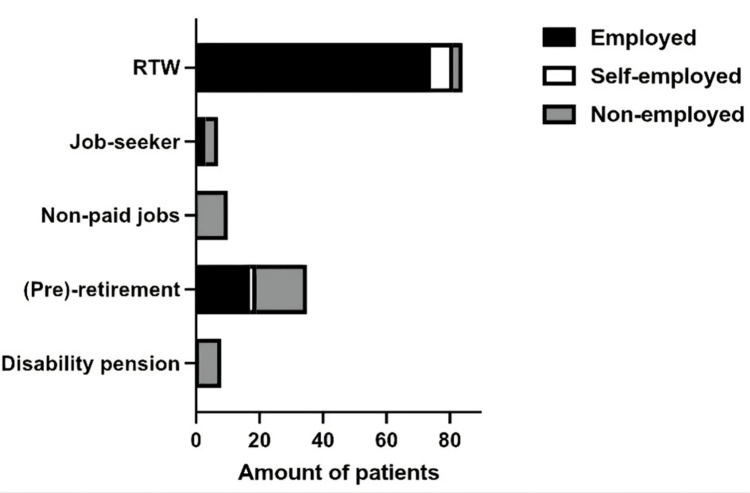
Number of patients with work status after surgery. RTW: return to work

Prior to surgery, 96 patients (66%) were employed, nine (6%) were self-employed, and 40 (28%) were non-employed. Among employed patients, 77% returned to full work at a mean of 7.4 ± 4.4 weeks. Self-employed patients showed a similar full RTW rate of 78%. Detailed RTW outcomes and timing are provided in Appendix C, and postoperative work status distribution is illustrated in Figure 2.

Occupational groups and RTW

RTW rates varied across occupational groups. Full RTW was most common in pedagogical, administrative, care, and welfare professions, whereas technical and physically demanding jobs often required lighter duties or reduced hours. Detailed numbers and percentages are shown in Table 2.

**Table 2:** Numbers of patients in various occupational groups according to the ROA-CBS 2014 classification of work prior to surgery and full and partial RTW. Small occupational groups were combined to avoid misleading percentages, retaining larger groups to highlight meaningful trends. RTW: return to work; ICT: Information and Communication Technology; ROA-CBS: Research Centre for Education and the Labour Market-Statistics Netherlands

	Prior to surgery		Full RTW		Lighter duties/fewer hours	
Occupational groups	N	%	N	%	N	%
Pedagogical and Educational	9	6	5	56	2	22
Creative, ICT, Linguistic, Other	9	6	5	56	2	22
Commercial and Administrative	32	22	20	63	2	6
Managers and Public Administration	15	10	10	67	3	20
Technical and Transport	27	19	11	41	7	26
Care and Welfare	22	15	9	41	4	18
Service and Other	21	14	9	43	6	29
Job unknown/non-existent	23	16	4	17	2	9
Total	145	100	65	45	27	19

Perceived factors influencing RTW

Patients reported several factors influencing RTW. The most frequently reported facilitators were social contacts at work (n = 47; 32%) and intrinsic motivation or job satisfaction (n = 54; 37%). Commonly reported barriers included physical complaints (n = 27; 19%), physically demanding work (n = 14; 10%), and limited mobility (n = 10; 7%). To facilitate earlier RTW, patients most often suggested more physiotherapy (n = 14; 10%), better guidance during the reintegration process (n = 14; 10%), and temporary adjustment of working conditions (n = 14; 10%). Detailed results are presented in Appendix D.

Predictors of RTW

A binary logistic regression analysis was conducted in 106 patients with complete data to identify predictors of RTW after THA. The model included sex, age, ASA classification, employment type, postoperative EQ-5D index, and postoperative contact with OP or social security services. The model showed acceptable fit and significantly improved prediction compared with the null model. Sex was the only significant predictor of RTW, with women having higher odds of not returning to work compared with men (odds ratio (OR) 3.74, 95% confidence interval (CI) 1.29-10.84). Age, ASA classification, employment type, postoperative EQ-5D index, and postoperative contact with OP or social security services were not significantly associated with RTW. The full logistic regression model for predictors of RTW after THA is presented in Appendix E.

RTW timing analysis

**Figure 3:**
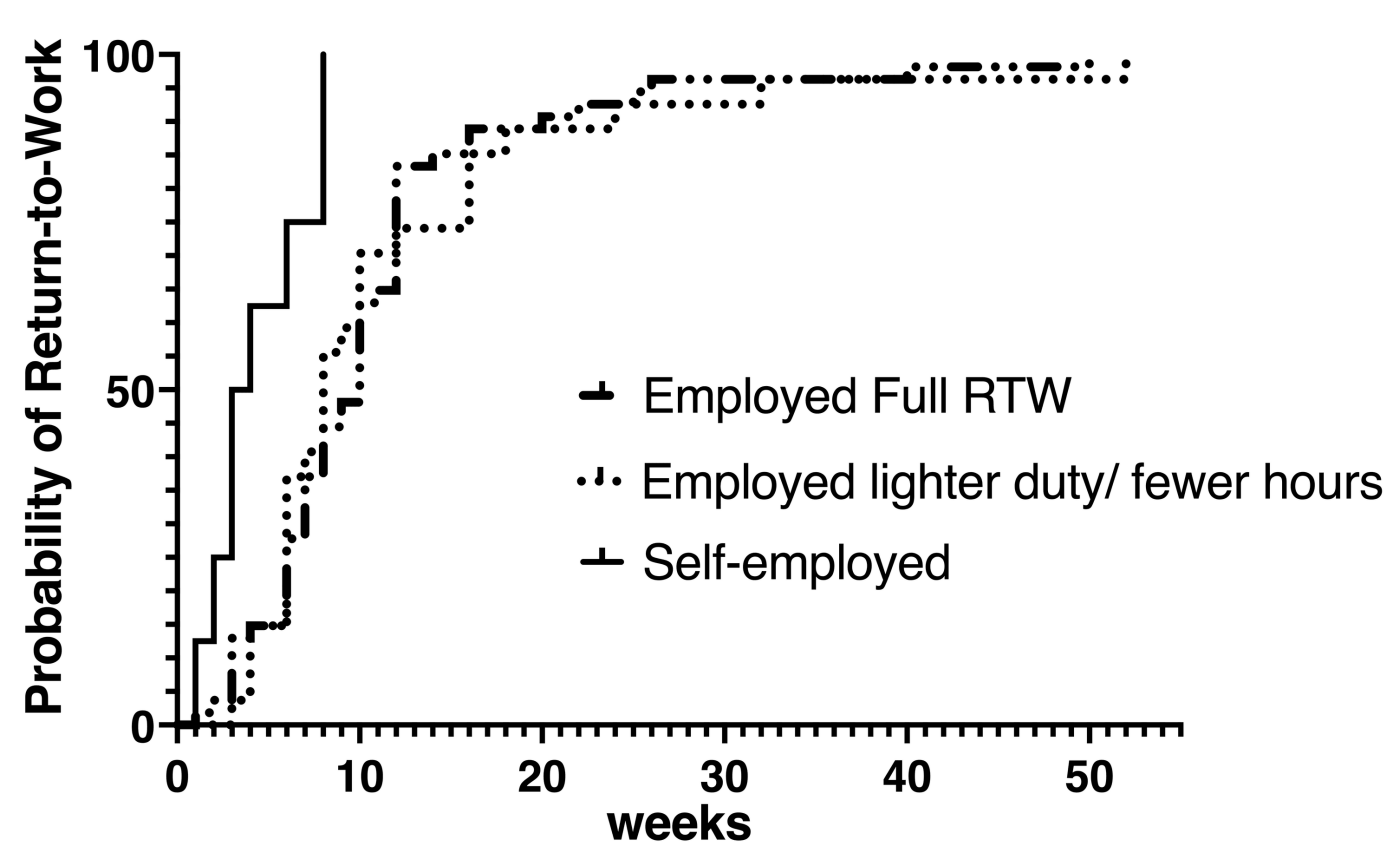
Reverse Kaplan-Meier analysis of RTW in weeks among employed patients. RTW: return to work

A reversed Kaplan-Meier analysis was performed on 105 patients working prior to surgery, stratified by employment type and RTW category (full RTW vs. lighter duties/reduced hours). Most patients RTW within the first 12-weeks, with remaining patients returning between 12 and 52 weeks postoperatively. This analysis is shown in Figure 3.

## Discussion

This study provides valuable insight into the factors influencing the time to RTW following THA. The findings indicate that the majority of patients resumed work within the first year, with a mean RTW time of 12 weeks postoperatively. Overall, 6% of patients (n = 8) did not RTW and were receiving disability benefits more than two years after surgery. Positive workplace interactions and job satisfaction emerged as key facilitators of successful RTW, whereas physically demanding occupations and persistent postoperative symptoms were associated with delayed RTW.

The present study extends follow-up to 104 weeks, exceeding the duration of earlier studies, and shows that patients who returned to work after surgery generally remained employed at two years. RTW rate in the second year is relatively low, with only one patient returning to work. A previous Dutch study reported that 13% of patients partially returned to work, while 7% did not return within one year following THA [18]. The current findings align with those results, confirming that the majority of patients return to work within the first postoperative year. This cohort resides in a region characterized by lower average health and SES compared to national levels [19]. Although no significant differences were observed in RTW outcomes between employed, unemployed, and self-employed individuals, further research is warranted to assess the potential long-term impact of SES on sick leave duration and disability benefit dependency. Integrating SES into the evaluation of THA outcomes underscores the importance of recognizing regional disparities, which may influence recovery trajectories, RTW success, and overall quality of life. Addressing these factors in clinical practice may enable more equitable care and improved outcomes across all SES groups.

In the multivariable logistic regression, sex was the only significant predictor of RTW, with women having nearly fourfold higher odds of non-RTW compared with men. This new finding highlights sex-specific disparities in recovery and work reintegration. Several mechanisms may contribute, including lower preoperative functional status, greater postoperative pain, slower recovery trajectories, and challenges balancing responsibilities, including household and rehabilitation [20-22]. Occupational sector differences, such as higher representation of women in physically demanding or care-related professions, may further exacerbate delayed RTW.

Contrary to some prior research, age, ASA classification, employment type, postoperative EQ-5D index score, and contact with occupational or social security services did not significantly predict RTW. This suggests that uncomplicated postoperative recovery may diminish the barrier effects of baseline comorbidity or job demands. It also indicates that other unmeasured factors, such as employer flexibility, workplace social support, and job autonomy, may be important determinants of RTW.

Previous studies have identified physical residual complaints, older age, and self-employment as factors negatively affecting RTW following THA [9,23,24]. Consistent with these findings, 28% (n = 27) of patients in the present study reported physical complaints as a barrier to RTW. Conversely, this study also highlights the positive role of intrinsic motivation and favorable psychosocial working conditions, such as job satisfaction and the social aspect of work, reported by approximately 25% of participants, as facilitators of successful RTW, aligning with prior research [25,26]. Interestingly, self-employed individuals RTW more rapidly than salaried employees [27], possibly due to financial pressures associated with the absence of sick leave benefits. Money-driven factors may influence the time to start reintegration or the length of the reintegration process.

A previous study has advocated implementing work-oriented care and targeted interventions to support RTW following THA. While a high proportion of patients in the present study successfully resumed work within one year, some did not return to work in the second year, highlighting the need for further evaluation of the effectiveness of such care. Earlier consultation with an OP does not appear to be beneficial [28]. Nevertheless, patients continue to express a need for practical guidance and enhanced exercise therapy. Early identification of individuals at risk for delayed or failed RTW should be prioritized to optimize outcomes and tailor support accordingly.

The logistic regression model explained approximately one quarter of the variance in RTW (Nagelkerke R^2^ = 0.274); however, this explanatory power was attributable solely to sex. Although the model showed high sensitivity (96%), specificity was low (21%), indicating limited ability to accurately identify individuals at risk of non-RTW. Consequently, the model has limited practical predictive utility, particularly for clinical decision-making. These findings are consistent with previous RTW prediction research, which also reports modest explanatory power and persistent challenges in predicting non-RTW [8,9,29,30]. Therefore, the model should not be interpreted as a standalone clinical prediction tool.

Strengths of this study include the detailed occupational classification, integration of clinical and vocational variables, and the use of validated measures such as the EQ-5D. This study has several limitations that may restrict the generalizability of its findings. The research was conducted in a peripheral teaching hospital and included a relatively small patient cohort. Generalization to other hospitals or countries with different healthcare systems may be limited. Furthermore, due to the retrospective study design and the timing of the questionnaire distribution postoperatively, patient responses may have been influenced by their current health status, potentially affecting the accuracy of the reported outcomes. Another limitation is the limited insight into work environment factors such as flexibility and autonomy, which may influence RTW outcomes. Selection bias may have occurred, as the reasons for non-participation among eligible patients were unknown, potentially affecting the results. In addition, missing data and the descriptive use of SES, which was not included as a predictor in the multivariable model due to limited variation within the sample, may have influenced the findings. Future studies with larger and more socioeconomically diverse cohorts should further examine the role of SES in RTW after THA.

## Conclusions

The findings indicate that the majority of patients returned to work within the first year following THA and remained employed at the 104-week follow-up. Patients reported that increased access to exercise therapy and structured guidance, alongside temporary modifications in work conditions and responsibilities, could have further supported their RTW. Among the demographic, clinical, and occupational predictors examined, female sex was the only independent predictor of non-RTW. These findings highlight important implications for clinical practice, as clinicians should be aware that women may require enhanced postoperative support, earlier vocational counseling, or closer follow-up to facilitate RTW.

The growing need to carry out THA will likely continue. Combined with an increasing number of patients who work at an advanced age, the importance of RTW will continue to grow. Considering the above, it is desirable to conduct additional, large-scale research with greater focus on the working-age population. Future research should incorporate detailed psychosocial measures, employer factors, and longitudinal follow-up to develop more accurate prediction models and targeted interventions to support work reintegration after THA.

## Appendices

Appendix A

**Table 3:** Return to Work Questionnaire (English version; original in Dutch).

Question					
1. In the 3 months prior to the operation, did you perform paid work?					
	a. Yes				
	b. No, I was				
	i. Retired				
	ii. Housewife/househusband				
	iii. Unemployed / Job seeker				
	iv. Unable to work due to complaints related to the joint to be replaced				
	v. Unable to work due to other health problems				
	vi. Other __________________				
2. After the operation, did you have contact with your occupational physician?					
	a. Yes				
	b. No				
3. After the operation, did you have contact with the Dutch National Institute for Employee Benefits Schemes?					
	a. Yes				
	b. No				
Describe the work you did PRIOR to the joint replacement surgery. If you had more than one job, describe the job in which you worked the most hours.					
4. My position or type of work was ______________________					
5. I worked					
	a. As an employee				
	b. As a self-employed worker				
6. I worked ______ hours per week.					
7. Indicate how often the following tasks applied to your work by selecting one of the following options:					
		Not or rarely required	Sometimes required	Frequently required	Continuously required
	Sitting				
	Standing				
	Walking				
	Squatting				
	Kneeling/Crawling				
	Climbing				
	Operating pedals with the legs				
	Lifting items from the ground				
	Lifting/pushing/pulling 5 kg				
	Lifting/pushing/pulling 10 kg				
	Lifting/pushing/pulling 20 kg				
	Lifting/pushing/pulling 45 kg				
8.If you are not currently employed, what was the last job you performed?					
9.If you are not currently employed, how many hours per week did you work?					
10. Since when have you not been employed?					
11. What is the reason you are no longer employed?					
	a. This operation				
	b. Other illness, namely _________________________________________________				
	c. Accident				
	d. Personal choice				
	e. Other, namely ______________________________________________________				
12. If you are not employed, do you receive (partial) benefits, and if so, which?					
	a. No, I do not receive any benefits				
	b. Yes, I receive benefits, namely				
	i. Benefit from the Work and Income (Capacity for Work) Act				
	ii. Benefit from the Disability Insurance Act				
	iii. Benefit from the Disability Benefits for Young Disabled Persons Act				
	iv. Sickness Benefit Act				
	v. Benefit from the Social assistance				
	vi. Other, namely ______________________________________________				
13. PRIOR to the operation, after how long did you expect to return to work?					
	______ weeks after the operation				
14. Where did you receive information or advice about returning to work after your operation?					
15. After your joint replacement surgery, did you return to work?					
	a. Yes, approximately ____ weeks after the operation				
	b. No, due to __________________________________________				
16. Can you indicate which factors would have helped you return to work sooner?					
17. What motivated you the most to return to work?					
18. What hindered you the most from returning to work?					
	If you have not yet returned to work, you do not need to answer the following questions.				
19. When you returned to work, you returned to:					
	a. The same number of hours and the same duties as before (prior to the joint replacement surgery)				
	b. Lighter duties				
	c. Fewer hours				
20. Looking back, do you feel that you returned to work:					
	a. At the right time				
	b. Too early				
	c. Too late / you feel that you could have returned earlier				

Appendix B

**Table 4:** Demographics and PROMs of the total cohort. PROMs: patient-reported outcome measures; SD: standard deviation; BMI: body mass index; ASA: American Society of Anesthesiologists classification; SES: socioeconomic status; EQ-5D: EuroQol Five Dimensions; VAS: Visual Analogue Scale; OHS: Oxford Hip Score

Characteristic	N	Mean ± SD or n (%)
Patients approached	381	-
Responded	182	53.8
Completed questionnaire	145	38
Age (years)	145	58.7 ± 5.6
Female sex	145	74 (51)
BMI (kg/m^2^)	145	28.4 ± 4.6
Time since surgery (years)	145	4.2 ± 0.8
Employment status	145	-
Employed	96	66
Self-employed	9	6
Non-employed	40	28
ASA classification	145	-
I	42	29
II	98	68
III	5	3
SES score	143	-0.05 ± 0.19
EQ-5D index Pre-op	145	0.752 ± 0.055
EQ-5D index Post-op	145	0.817 ± 0.34
EQ-5D VAS Pre-op	145	59.8 ± 19.8
EQ-5D VAS Post-op	145	78.2 ± 15.8
OHS Post-op	145	38.3 ± 9.8

Appendix C

**Table 5:** RTW outcomes and timing. Percentages are calculated within patients employed or self-employed preoperatively. Partial RTW includes lighter duties or reduced hours. RTW: return-to-work

RTW category	N	%	Mean time to RTW (weeks ± SD)
Employed, full RTW	74	77	7.4 ± 4.4
Self-employed, full RTW	7	78	7.2 ± 3.8
Partial RTW/lighter duties	27	19	11.6 ± 7.5
Disability benefits (after 104 weeks)	8	6	-

Appendix D

**Table 6:** Factors influencing RTW. Percentages are based on the total study population; patients could report multiple factors. RTW: return-to-work

Factor category	Factor	N	%
Positive contributors	Social contacts at work	47	32
	Intrinsic motivation/job satisfaction	54	37
Negative contributors	Physical complaints	27	19
	Physically demanding work	14	10
	Limited mobility	10	7
Patient suggestions	More physiotherapy	14	10
	Better guidance during reintegration	14	10
	Temporary adjustment of working conditions	14	10

Appendix E

**Table 7:** Logistic regression analysis of predictors for RTW after THA (n = 106). RTW: return-to-work; THA: total hip arthroplasty; OR: odds ratio; CI: confidence interval; ASA: American Society of Anesthesiologists classification; OP: occupational physician. Significant predictors are in bold. Model χ^2^ = 22.26, p = 0.014; Nagelkerke R^2^ = 0.27; Hosmer-Lemeshow p = 0.206.

Predictor	B	SE	Wald	p-value	OR (Exp(B))	95% CI
Sex (female vs. male)	1.318	0.544	5.87	0.015	3.74	1.29-10.84
ASA I vs. II-III	-0.325	0.527	0.38	0.538	0.72	0.26-2.03
Age (years)	-0.028	0.042	0.44	0.509	0.97	0.90-1.06
Post-op contact OP (yes vs. no)	-0.581	0.733	0.63	0.427	0.56	0.13-2.35
EQ-5D index post-op	-0.980	0.855	1.32	0.251	0.38	0.07-2.00
Constant	-42.110	46209.918	0.000	0.999	-	-
